# *Mentha pulegium* L.: A Plant Underestimated for Its Toxicity to Be Recovered from the Perspective of the Circular Economy

**DOI:** 10.3390/molecules26082154

**Published:** 2021-04-08

**Authors:** Lucia Caputo, Laura Cornara, Francesco Maria Raimondo, Vincenzo De Feo, Stefano Vanin, Marcella Denaro, Domenico Trombetta, Antonella Smeriglio

**Affiliations:** 1 Department of Pharmacy, University of Salerno, via Giovanni Paolo II, 132, 84084 Fisciano (SA), Italy; lcaputo@unisa.it (L.C.); defeo@unisa.it (V.D.F.); 2 Department of Earth, Environment and Life Sciences (DISTAV), University of Genova, Corso Europa, 26, 16132 Genova, Italy; laura.cornara@unige.it (L.C.); stefano.vanin@unige.it (S.V.); 3 PLANTA/Autonomous Center for Research, Documentation and Training, Via Serraglio Vecchio, 28, 90123 Palermo, Italy; cescoraimondo@gmail.com; 4 Department of Chemical, Biological, Pharmaceutical and Environmental Sciences, University of Messina, Via Giovanni Palatucci, 98168 Messina, Italy; marcella.denaro@unime.it (M.D.); antonella.smeriglio@unime.it (A.S.)

**Keywords:** *Mentha pulegium* L., micromorphology, trichomes, essential oil, phytochemistry, phytotoxicity, α-amylase, eco-compatibility

## Abstract

The aim of the study was to investigate the micromorphology of *Mentha pulegium* leaves and flowers harvested in three different Sicilian (Italy) areas with peculiar pedo-climatic conditions, and to characterize the phytochemical profile, the phytotoxic activity, and the eco-compatibility of their essential oils (EOs) for potential use as safe bioherbicides. Light microscopy (LM) and scanning electron microscopy (SEM) highlighted that *M. pulegium* indumentum consists of non-glandular and glandular trichomes of different types. Peltate trichomes of plants from the different sites showed few significant differences in dimension and abundance, but they were characterized by a surprisingly high number of secretory cells both in leaves and flowers. Phytochemical analyses showed that oxygenated monoterpenes were the most abundant class in all the EOs investigated (92.2–97.7%), but two different chemotypes, pulegone/isomenthone and piperitone/isomenthone, were found. The complex of morphological and phytochemical data indicates that soil salinity strongly affects the expression of the toxic metabolite pulegone, rather than the EO yield. Phytotoxicity tests showed a moderate activity of EOs against the selected species as confirmed by α-amylase assay. Moreover, the low toxicity on brine shrimp provided a rationale for the possible use of investigated EOs as eco-friendly herbicides.

## 1. Introduction

*Mentha pulegium* L., belonging to the Lamiaceae family and commonly named pennyroyal [1], is an aromatic and tomentose perennial herb widespread in Europe, Middle East, and North Africa, which grows in alluvial plains, riparian habitats, and freshwater wetlands [2]. It presents stems procumbent to ascending, narrowly ovate or elliptic leaves, and flowers in well-spaced verticillasters in the leaf axils [3]. *M. pulegium* has been well known since ancient times, from Greek, Roman, and Medieval cultures, for its culinary uses and medicinal properties, such as emmenagogue and abortifacient effects [4,5], as well as for treatment of gastrointestinal ailments and skin itching [6]. Nowadays, *M. pulegium* commercialization includes uses as food and drink flavoring, Pennyroyal teas for cold relief, coughs, kidney problems, and headaches [7]. Despite these applications, this plant is well-known for its toxicity to humans, especially due to its essential oil (EO).

It is well-known that a small dose (<10 mL) of *M. pulegium* EO can cause nausea, vomiting, abdominal pain, and dizziness, whereas a multiorgan failure, which can even lead to death, can occur by the consumption of a larger dose [8,9,10,11]. The compound mainly responsible for the toxic effects of *M. pulegium* is pulegone, which confers to the plant the typical peppermint flavor which, generally, is very abundant in its EO. This compound, once ingested, can act as an enzyme inhibitor and it has a marked organotropism for the kidney and liver [12]. Despite no current toxicokinetic studies are available on humans, someone has investigated the effects of pulegone on other mammals. These studies demonstrated that pulegone, once ingested, is broken down by the liver and reacts to form multiple toxic and potentially harmful metabolites such as menthofuran, piperitenone, piperitone, and menthone [12]. In vivo studies carried out by the National toxicology program [13] demonstrated the involvement of pulegone in the onset of urinary bladder disease in female rats and liver cancer in male ones as well as unusual kidney lesions and hyaline glomerulopathy in both female and male rats. However, a recent assessment of the Expert Panel of the Flavor and Extract Manufacturers Association (FEMA) affirmed the status generally recognized as safe (GRAS) to pennyroyal oil [14]. This assumption was supported by the use levels that do not saturate the pathways of metabolism and excretion, as well as by an adequate margin of safety between time exposure and no-observed-adverse-effect levels in animals during short and long-term toxicity studies. Moreover, *M. pulegium* EO did not result in genotoxic or mutagenic effects [14].

*M. pulegium* EO is commonly used as insecticides and pest repellents for pets and humans, and to keep fleas, mosquitos, and gnats away [15].

Recently, the potential damage of chemical pesticides and herbicides to human health has become a high concern and there is an increasing interest in new bio- and eco-sustainable formulations as alternative strategies. Although the pesticide activity of *M. pulegium* has been demonstrated, no studies are currently available regarding its phytotoxic activity and toxicity on aquatic organisms.

Moreover, it is well-known that the plant’s growth conditions largely influence the secondary metabolites expression and consequently the biological activity and toxicity.

Considering this, the aim of the present study was to investigate the micromorphological features as well as the phytochemical profile, phytotoxic activity, and toxicity of EOs obtained by leaves and flowers of *M. pulegium* plants grown in three different areas of Sicily (Italy) with particular pedo-climatic conditions.

## 2. Results and Discussion

### 2.1. Micromorphological Studies and Essential Oil Yield

**Figure 1:**
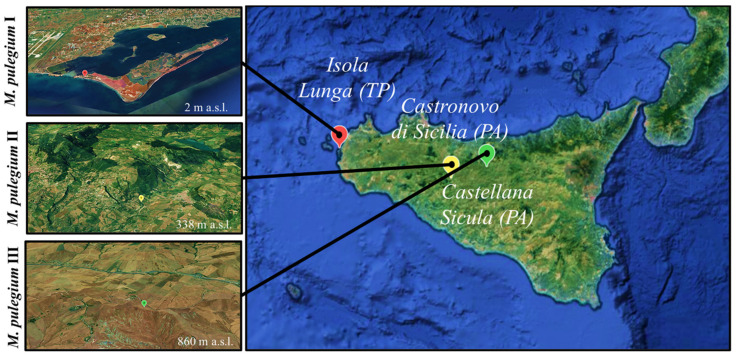
Collection sites identified by Google Earth (https://www.google.it/earth/, accessed data 15 February 2021) using the GPS coordinates.

Micromorphological analyses were performed on mature leaves and flowers of *M. pulegium* plants, collected at a full flowering stage in three different sites of Sicily (MP-I, MP-II, and MP-III) as shown in Figure 1.

The glandular trichomes show the common features described in the Lamiaceae [16], with two main types, peltate and capitate, as previously reported in other *Mentha* species [17,18,19] including *M. pulegium* [18,20].

**Figure 2:**
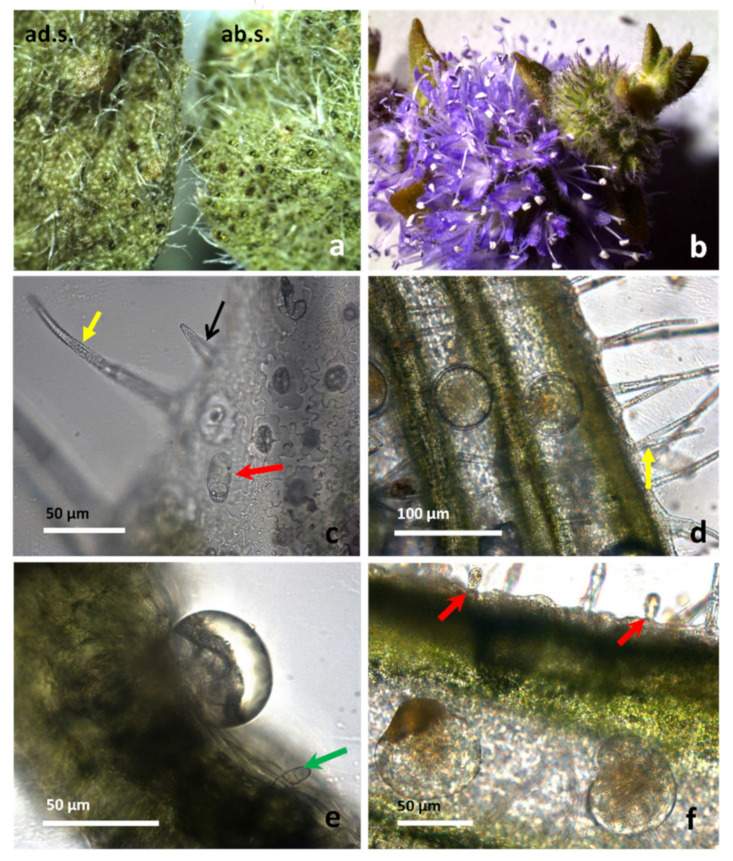
*M. pulegium* leaves (**a**, **c** and **e**) and flowers (**b**, **d** and **f**). (**a**) Comparison between adaxial (ad.s.) and abaxial (ab.s.) surfaces of mature leaves, showing a higher density of peltate trichomes on the abaxial side. (**b**) Inflorescence. Different kinds of trichomes are visible on the leaf (**c**,**e**) and on the calyx (**d**,**f**). (**c**) A short unicellular trichome is indicated by black arrows, a long multicellular uniseriate trichome by a yellow arrow, and a capitate type I trichome by a red arrow. (**d**) On the flower calyx, many peltate trichomes are scattered between the ribs, while many long, non-glandular trichomes are also visible (yellow arrow). (**e**) Transversal section of a leaf with a peltate trichome showing a large subcuticular space filled with EO, and a capitate type II trichome (green arrow).

**Figure 3:**
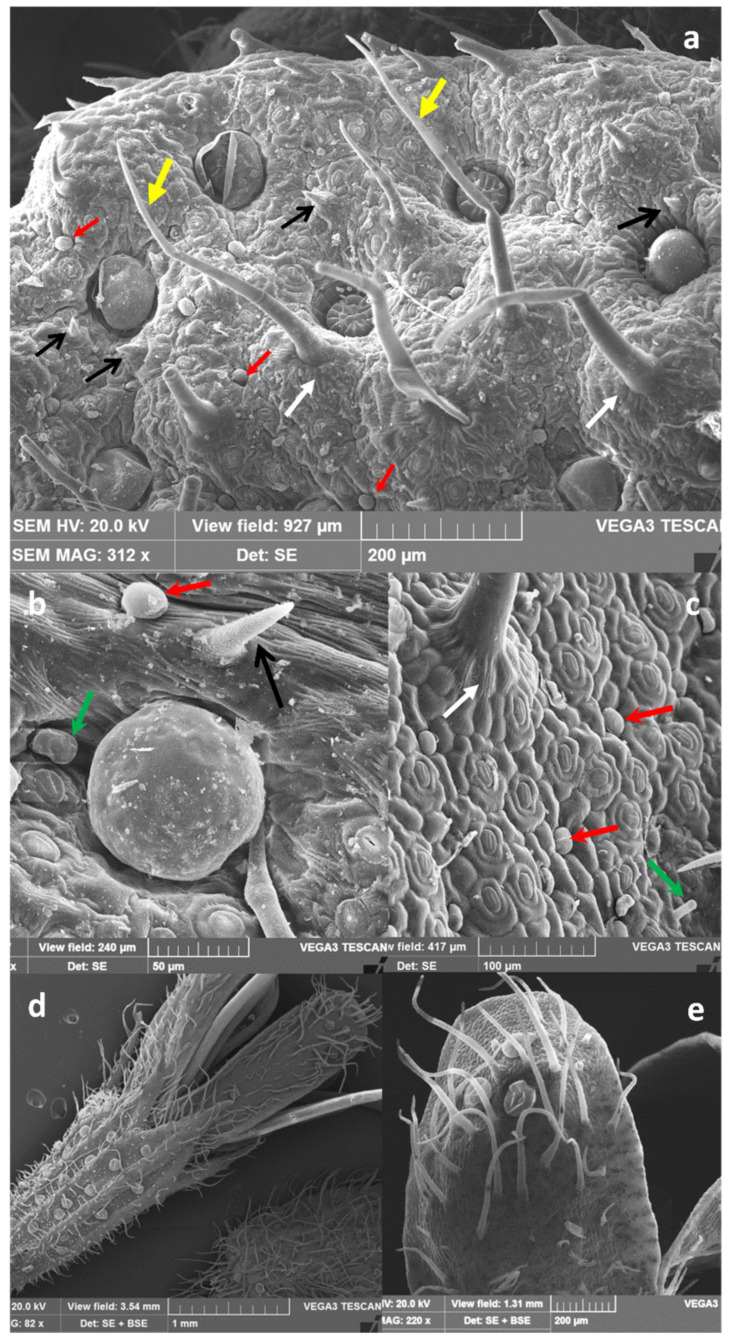
*M. pulegium* leaves (**a**–**c**) and flowers (**d**,**e**). Non-glandular trichomes and glandular trichomes on the leaf surface of mature leaves are shown (**a**–**c**); (**a**), capitate type I trichomes are indicated by red arrows, and capitate type II ones by green arrows; black arrows indicate short unicellular trichomes, yellow arrows indicate long multicellular uniseriate trichomes, while around their base, epidermal cells arranged in a circle to form a pedestal are shown by white arrows. (**d**) Long multicellular, uniseriate non-glandular trichomes are visible on the calyx, together with many peltate trichomes arranged in rows between the ribs. (**e**) On the outer face of the petal apex, multicellular trichomes appear thin, long, and lean toward the apex, while only a very few peltate trichomes can be found.

Our observations, carried out by light microscopy (LM) and scanning electron microscopy (SEM), confirmed that *M. pulegium* indumentum consists of non-glandular trichomes and glandular trichomes covering both epidermises of mature leaves (Figure 2a,c,e; Figure 3a–c) and flowers (Figure 2b,d,f; Figure 3d,e).

**Figure 4:**
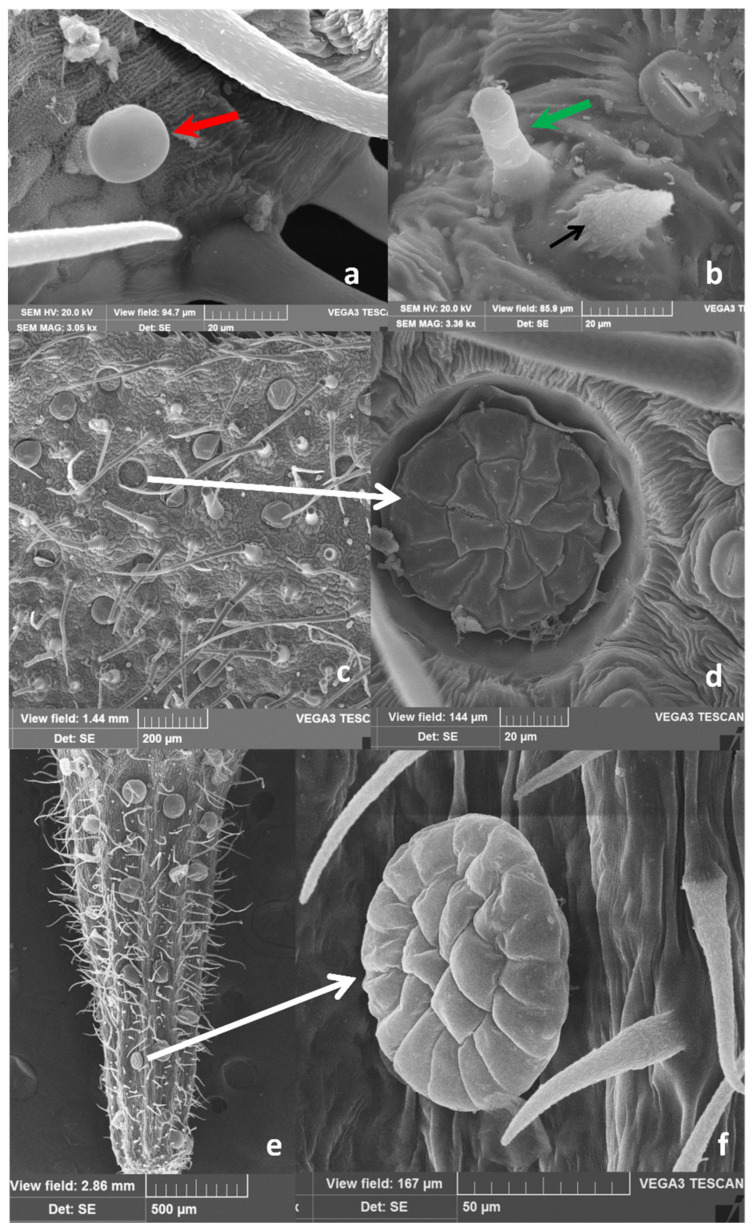
*M. pulegium*, detail of the different kinds of trichomes present on the leaf (**a**,**b**): capitate type I (**a**, red arrow); capitate type II (**b**, green arrow), and short non-glandular trichome (**b**, black arrow). (**c**,**d**) (leaf) and (**e**,**f**) (flower calyx): peltate trichomes on the leaf (**c**) and calyx (**e**). At higher magnification a high number of secretory cells, arranged in two circles within the head, can be observed (**e**,**f**, white arrows).

According to data referred to by Rodrigues et al. [18], different types of non-glandular trichomes were observed: (i) short unicellular trichomes, with a swollen basal epidermal cell and acute apices, showing a warty surface, which was scattered on leaves and calyx (indicated by black arrows in Figure 2c, Figure 3a,b, and Figure 4b); (ii) medium to long multicellular and uniseriate trichomes, with a likely protective role, showing a slightly warty surface and acute apices (Figure 2c,d, and Figure 3a, yellow arrows).

These trichomes are supported by five to eight epidermal cells, arranged in a circle around the base and forming a pedestal (Figure 3a,c, white arrows), and are spread on leaf surfaces and calyx (Figure 2, Figure 3 and Figure 4). On the petal apex outer face, multicellular uniseriate trichomes appear thin, long, and leaned toward the apex (Figure 3d,e). Two types of capitate trichomes were also found: type I with a basal cell, a short stalk cell, and a large, round to oval, one-celled secretory head (indicated by red arrows in Figure 2c,f, Figure 3a–c, and Figure 4a). Capitate type II showed a basal cell, a long stalk cell, and a smaller unicellular secretory head (Figure 2e, Figure 3b,c, and Figure 4b, green arrows). According to data reported by Werker et al. [16], and Ascensão and Pais [21], the capitate glandular trichomes of the Lamiaceae have a limited storage capacity, and their secretion mainly consists of a complex mixture of polysaccharidic substances and minor amounts of lipids and proteins. In *M. pulegium* peltate glandular trichomes are spread on leaves, and more abundant on the abaxial epidermis (Figure 2a; Figure 3a; Figure 4c) and reproductive organs, where they appear very abundant on the calyx, arranged in almost regular rows between the ribs (Figure 2d,f; Figure 3d; Figure 4e). On the contrary, they are present in negligible quantity (3–5) on the apex of outer petal surfaces (Figure 3d,e).

The peltate trichomes consist of a basal cell, a short stalk, and a broad head, with a variable number of secretory cells, arranged in two circles. Secretory cells are surmounted by a large subcuticular space containing the bulk of EO, as previously noted in peppermint plants [22,23]. In the wild populations of *M. pulegium* from Sicily examined here, we found a surprisingly high number of secretory cells within peltate trichomes: 4–7 in the inner circle and 8–13 in the outer one, giving rise to 12–18 celled trichomes in the expanded mature leaves (Figure 4c,d, at higher magnification, white arrow). In the flower, peltate trichomes are abundant on the calyx where they appear arranged in rows between the ribs (Figure 2d,f; Figure 3d; Figure 4e), while only a few are present on the outer face of petals (Figure 4d–e). In reproductive organs, the number of secretory cells within these trichomes is higher with respect to that found on leaves, reaching up to 20–22 cells (Figure 4f). Considering that peltate trichomes only secrete lipophilic substances, being the main sites of EO secretion and that they are mainly abundant on leaves and flower calyx, we calculated their density and head dimension on adaxial and abaxial surfaces of mature leaves and on the calyx. The results are summarized in Table 1. Peltate trichome density is from 3 to 6 folds higher in the leaf abaxial surface (ab.s.) with respect to the adaxial surface (ad.s.). In the flower calyx, a higher density of trichomes is present in MP-II with respect to MP-I. The trichome diameter is always significantly higher in the flower calyx with respect to the leaf. In addition, the head diameter is significantly higher in MP-I leaves with respect to MP-III ones. In conclusion, the trichomes of plants from the different Sicilian sites show few significant differences in dimension and abundance.

Our data concerning differences in peltate trichomes density between the two-leaf epidermis are consistent with those of Karray-Bouraooui et al. [20] and Rodrigues et al. [18], showing a more abundant presence on the leaf abaxial surface. In addition, wild populations of *M. pulegium* growing in Portugal [18] showed that the head of peltate trichomes located on reproductive organs were bigger, compared to those of mature leaves, in agreement with our observations. However, these authors reported a number of 12 secretory cells forming the head disc in mature leaves and up to 16 in reproductive organs. Conversely, in plants from Sicily secretory cells consist of up to 18 cells in the peltate trichomes of mature leaves and up to 22 in those of reproductive organs.

Also, the density of peltate trichomes in the expanded mature leaves was lower in plants from Portugal compared to those of Sicily. Density data for Portugal plants are 2.7 and 3.9 trichomes/mm^2^ in the adaxial and abaxial surface, respectively, whereas in our plants from three sites of Sicily these values are 2.6–5.0 and 15.9–21.6 trichomes/mm^2^ in the adaxial and abaxial surface, respectively (Table 1). Our data are similar to those reported for 7 different species of *Mentha* from China by Yu et al. [19], where the density of peltate trichomes on the leaf abaxial surface was shown to vary from a minimum of 9.7 in *M. rotundifolia*, to a maximum of 19.9 trichomes/mm^2^ in *M. piperita*. However, in these species, peltate trichomes had a small number of secretory cells (8–16).

For the species from China, the EO yield at the blooming stage was reported to range from 0.7 to 1.4% (w/d.w.), according to data of Rodrigues et al. [18], reporting for *M. pulegium* at a full flowering stage has an EO yield of about 1.6% (w./d.w.). On the contrary, the EO yield of the three *M. pulegium* plants from Sicily (Figure 1) was higher ranging from 2.7 to 3.2% (v/d.w) for leaves (MPL), and from 2.6 to 2.8% (v/d.w) for flowers (MPF), without significant differences between leaves and flowers (Table 1). The average EO yield fits perfectly within the range previously reported for other *Mentha* spp. (1.0–3.9%) by Hussain et al. [24], approaching the upper limit and confirming that mint plants, which grow in sunny places, characterized by high temperatures and low water supply, have the highest yield in EO [25].

In our study, the higher salinity of the clayey-saline depressions where MP-I grows, in comparison with the soil of MP-II and MP-III (Table 1), does not significantly affect peltate trichomes density, in contrast with observations of Karray-Bouraoui et al. [20]. These authors, however, studied plants obtained from seeds and grown under controlled conditions, in contrast with the wild growing conditions of our plants. Our data indicate that soil salinity influences the composition of the EO, rather than its yield or the density and distribution of peltate trichomes.

### 2.2. Essential Oil Composition

The phytochemical profile of the six EOs (MPLI, MPLII, and MPLIII, and MPFI, MPFII, and MPFIII) were investigated by gas-chromatography combined with flam ionization and mass spectrometry (GC-FID and GC-MS) analyses.

Table 2 shows the chemical composition of the *M. pulegium* EOs, listing compounds according to their elution order on the HP-5MS column (Agilent J&W, Santa Clara, CA, USA).

Oxygenated monoterpenes resulted in the most abundant class in all the EOs investigated ranging from 92.2 in MPLI to 97.7% in MPFIII followed by other compounds such as esters and ketones in MPLI and MPFI (4.8 and 3.5%, respectively) as well as in MPLIII and MPFIII (4.2 and 1.9%, respectively), and monoterpene hydrocarbons in MPLII and MPFII (2.6 and 4.0–44.0%, respectively). Thirty-six compounds were found in MPLI, with isomenthone and pulegone, which represent the most abundant compounds (34.0% and 56.8%, respectively). MPFI showed a phytochemical profile almost superimposable with which one of MPLI (isomenthone 30.9 and pulegone 60.2%) with 34 identified compounds. Fifteen and 29 compounds were identified in MPLII and MPFII EOs, whereas 33 and 9 compounds were found in MPLIII and MPFIII EOs, respectively. The phytochemical profile of these four EOs showed piperitone as the main constituent (88.5 and 88.0% in MPLII and MPFII, respectively; 80.3 and 89.4% in MPLIII and MPFIII, respectively), followed by isomenthone (7.2 and 5.3% for MPLII and MPFII, respectively; 13.0 and 8.0% for MPLIII and MPFIII, respectively).

Moreover, interestingly, pulegone was completely absent in MPLII, MPFII and MPFIII, and it was present in very low amount in MPLIII (0.2%). On the contrary, piperitone was found in very low amounts in both MPLI and MPFI EOs (0.5% and 0.6%, respectively).

The phytochemical profile of MPLI and MPFI EOs is in line with most of the previous studies, which showed pulegone as the main constituent of the *M. pulegium* EO (50.0%–73.3%) [26,27]. Moreover, pulegone/isomenthone ratios (60.3%/22.7% and 66.8%/34.8%) were comparable with the ones observed in MPLI (56.8%/34.0%) and MPFI (60.2%/30.9%), were found by Rodrigues and co-workers [18] and Fancello and co-workers [28], respectively.

On the contrary, some studies report a low to moderate content of this oxygenated monoterpene (2.3–21.3%) [29,30] and a higher expression of piperitone, ranging from 0.1 to 38.0% [7,29,31], without reaching, however, the values found in the present study for MPLII and MPLIII as well as for MPFII and MPFIII EOs (piperitone ≥ 80.3%).

**Figure 5:**
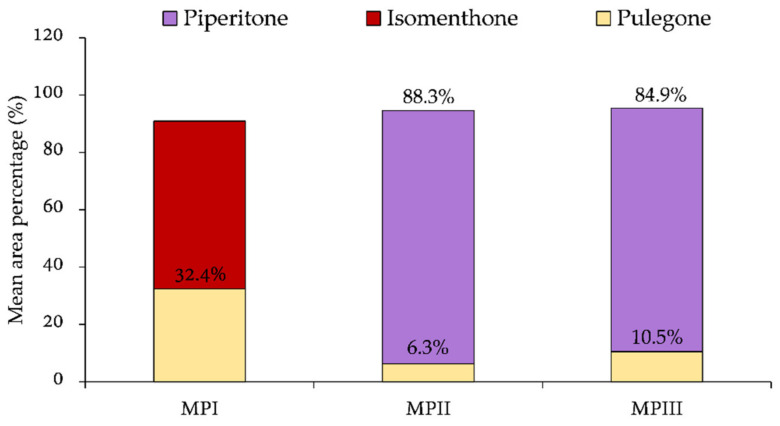
Chemotypes identified in *M. pulegium* plants collected in the three Sicilian sampling areas (I, II, and III). Percentage values reported in the graph bar refer to the average content of pulegone, isomenthone, and piperitone found in both EOs from leaves and flowers from each sampling point (see Section 3.2 for more details).

Results obtained in the present study demonstrated indirectly the influence of pedo-climatic conditions on secondary metabolites expression, which exert an important role in the phytochemical composition of *M. pulegium* EOs allowed to identify two different chemotypes (pulegone/isomenthone for MPLI and MPFI EOs, and piperitone/isomenthone for MPLII, MPFII, MPLIII, and MPFIII EOs) (Figure 5).

The main difference between the *M. pulegium* plant from site I and the other two (MP-II and MP-III) is that the first one grew up in clayey-saline sublittoral depressions occasionally affected by winter storms, whereas the MP-II and MP-III grew up in moist clay soils, in sites higher than 300 m a.s.l.

Considering this, the salinity and the altitude seem to play a pivotal role. Indeed, as described by Kasrati and co-workers [32], plants collected from low altitudes, such as MP-I, were subjected to stressful conditions preferring the pathway of pulegone and its derivative isomenthone, whereas higher altitudes lead to the lowest temperatures and humid conditions, in which plants are less stressed, preferring the pathway of piperitone. In addition, it has been demonstrated that salinity specifically increases the pulegone content of *M. pulegium* EO [20] corroborating the results obtained in the present study. However, although the altitude certainly represents a discriminating factor, the soil composition seems to exert a predominant role. Indeed, as demonstrated by Rodriguez et al. [18], who analyzed 14 EOs of *M. pulegium* coming from different areas of Portugal, even if the plants are sampled at different altitudes and the phytochemical profile of the EOs undergoes variations (decreasing the content of pulegone and isomenthone with respect to the content of piperitone and its derivatives), they always belong to the same chemotype (pulegone/isomenthone). These results confirm what has been observed in the present study: the particular phytochemical profile found in MPLI and MPFI is mainly due to the particular soil features of the sampling site, characterized by very high salinity. On the contrary, the phytochemical profile found in plants II and III is evidently characteristic of the Sicilian territory, which allows for the identification of peculiar and interesting chemotypes of *M. pulegium.*

### 2.3. Phytotoxic and Anti-α-Amylase Activity

The possible phytotoxic activities of EOs were tested on five dicotyledoneous (*Raphanus sativus* L., *Lactuca sativa* L., *Lepidium sativum* L., *Solanum lycopersicum* L., and *Portulaca oleracea* L.) and one monocotyledonous plant species (*Lolium multiflorum* Lam.).

**Figure 6:**
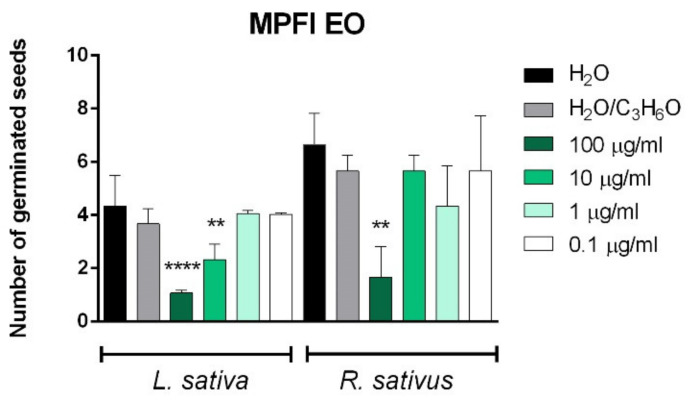
Phytotoxic activity of *M. pulegium* leaves (MPLI) and flowers (MPFI) EOs against germination of *P. oleracea*, and radical elongation of *L. sativa* and *R. sativus* 120 h after sowing. Results are the mean of three experiments ± standard deviation. ** *p* < 0.01, **** *p* < 0.0001 compared with control (ANOVA followed by Dunnett’s multiple comparison test).

**Figure 7:**
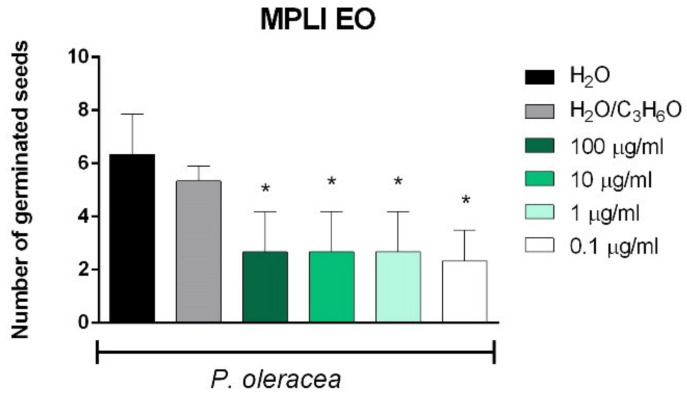
Phytotoxic activity of *M. pulegium* flowers (II) EO against the radical elongation of *L. sativa* (A) and germination of *R. sativus* (B) 120 h after sowing. Results are the mean of three experiments ± standard deviation. * *p* < 0.05compared with control (ANOVA followed by Dunnett’s multiple comparison test).

**Figure 8:**
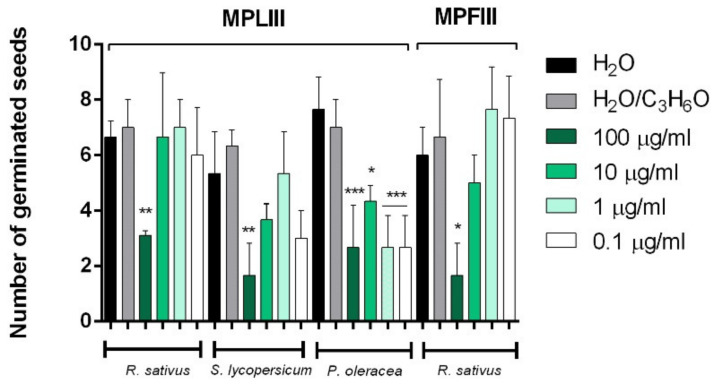
Phytotoxic activity of *M. pulegium* leaves (MPLIII) and flowers (MPFIII) EOs against germination of *P. oleracea, R.sativus,* and *S. lycopersicum* 120 h after sowing. Results are the mean of three experiments ± standard deviation. * *p* < 0.05, ** *p* < 0.01, *** *p* < 0.001, compared with control (ANOVA followed by Dunnett’s multiple comparison test).

**Figure 9:**
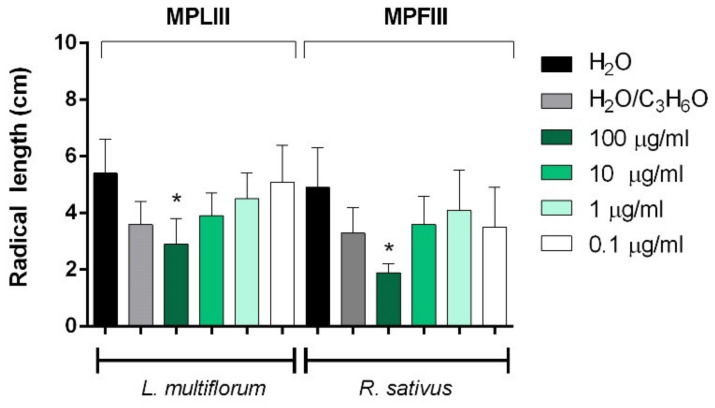
Phytotoxic activity of *M. pulegium* leaves (MPLIII) and flowers (MPFIII) EOs against the radical elongation of *L. multiflorum* and *R. sativus* 120 h after sowing. Results are the mean of three experiments ± standard deviation. * *p* < 0.05compared with control (ANOVA followed by Dunnett’s multiple comparison test).

Figure 6, Figure 7, Figure 8 and Figure 9 depict the phytotoxic activity of MPLI, MPFI, MPLII, MPFII, MPLIII, MPFIII EOs against germination of *R. sativus*, *P. oleracea*, *L. sativa*, and *S. lycopersicum* seeds and radical elongation of *R. sativus*, *L. multiflorum*, and *L. sativa* seeds.

Few variations in the chemical composition of the EOs could change the potential synergy between compounds and consequently the biological activities. In fact, even if MPLI and MPFI EOs have the same main constituents, their phytotoxic activity was different: MPLI EO was able to inhibit germination of *P. oleracea* seeds at all concentration used, whereas MPFI EO inhibited germination of *R. sativus* at the highest concentration used (100 µg/mL) and germination of *L. sativa* at concentrations of 100 µg/mL and 10 µg/mL (Figure 6).

Piperitone was the main constituent in the other four EOs studied (ranging from 80.3 to 89.4%). It is an oxygenated monoterpene similar to pulegone, biologically active against insects [33] as well as against human and plant pathogen fungi [34]. In this study, it seems to have moderate phytotoxic activity. Indeed, no seeds were affected both for germination and radical elongation by MPLII EO (see Supplementary Materials), whereas the MPFII EO inhibited, at 100 µg/mL, the radical elongation of *L. sativa* and the germination of *R. sativus* (Figure 7).

Moreover, MPFIII EO was active against radical elongation and germination of *R. sativus* at 100 µg/mL (Figure 8 and Figure 9). Instead, MPLIII EO was active against the radical elongation of *L. multiflorum* seeds at a concentration of 100 µg/mL (Figure 9) and inhibited germination of *P. oleracea* at all concentrations used (Figure 8). Moreover, the germination of *R. sativus* and *S. lycopersicum* was inhibited only at the highest concentration used (100 µg/mL) (Figure 8).

Few studies reported the possible phytotoxic activity of *M. pulegium* EOs. Kimbaris and coworkers [35] reported that *S. lycopersicum* was significantly affected by EOs derived from two populations of Grecian *M. pulegium*: one rich in piperitone (96.5%) and another in pulegone (74.4%). In our study, only the EO from MPFIII inhibited the germination of *S. lycopersicum* seeds at 100 µg/mL, although piperitone or pulegone are the main constituents in all our samples, this probably because in the previous study authors used higher doses of EOs (0.4 and 0.2 mg/mL). Considering this, our EOs seemed to be more phytotoxic than Grecian *M. pulegium* EOs.

Our results on *L. sativa* seeds agree with the results of Santana and collaborators [36], who showed that a Moroccan *M. pulegium* EO (pulegone, 88.2%) at a concentration of 5 µg/mL showed no significant phytotoxic effects on either *Lolium perenne* and *L. sativa*; indeed, our samples of MPFI and MPFII were active in germination or radical elongation of *L. sativa* seeds only at higher doses (100 µg/mL). No previous studies reported data of phytotoxic activity for *M. pulegium* EOs against *R. sativus*, *L. sativum*, *P. oleracea,* and *L. multiflorum*.

Changes which occur in the early stages of germination are due to various enzymes, which allow for the metabolism of starch, proteins, hemicellulose, lipids, and other storage material [37].

α-Amylase has an active role in the hydrolysis of the starch during seed germination and it is involved in the growth regulation [38]. In light of this evidence, in this study we also evaluated the possible influence of the six EOs on α-amylase activity.

The results are reported in Table 3 and showed that the EOs, which affected germination and/or radical elongation of tested seeds, are active also against α-amylase. MPFIII and MPLI EOs are more active than the other EOs with IC_50_ values of 638.9 µg/mL and 662.8 µg/mL, respectively.

The anti- α-amylase activity of MPFI, MPFII, MPLIII EOs are similar, with IC_50_ ranging from 785.3 to 1076.7 µg/mL; MPLII EO was inactive against α-amylase.

The variability in IC_50_ values may be attributed to the difference in starch content of the seeds of varieties considered, and to the involvement of other various enzymes in the germination process.

No previous studies reported the activity of EOs from *Mentha* spp. on α-amylase in association with phytotoxic activity. Figueroa-Pérez et al. [39] reported that *M. piperita* infusions showed a dose-dependent inhibitory activity of α-amylase with a maximum inhibition (45%) at a concentration of 60.0−80.0 mg/ mL.

### 2.4. Toxicity

The toxicological test on *Artemia salina* is a useful and rapid screening tool to evaluate the toxicity of the EOs, especially in the perspective of using them as new bio-herbicides. Indeed, through this assay, it is possible to highlight their eco-compatibility by investigating the toxicity on aquatic organisms, which are inevitably interested by the passage of EOs into underground aquifers.

**Figure 10:**
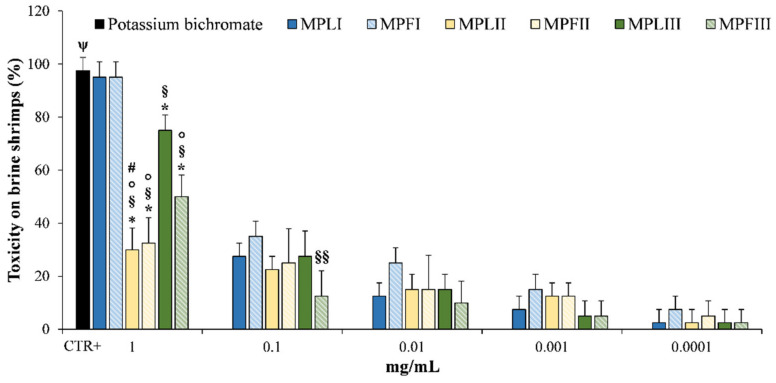
Toxicity on brine shrimps (*Artemia salina*) by EOs of *M. pulegium* leaves (MPLI, MPLII, and MPLIII) and flowers (MPFI, MPFII, and MPFIII) at 48 h. Potassium bichromate (50 μg/mL) was used as a positive control (CTR+). Results are the mean of three independent experiments ± standard deviation (*n* = 10). * *p* < 0.001 vs. MPLI; ^§^ *p* < 0.001 vs. MPFI; ^§§^ *p* < 0.05 vs. MPFI ° *p* < 0.001 vs. MPLIII; ^#^ *p* < 0.05 vs. MPFIII; ^ψ^ *p* < 0.001 vs. all sample concentrations except MPLI and MPFI.

*M. pulegium* EOs were tested at different concentrations ranging from 0.0001 to 1 mg/mL and results are shown in Figure 10.

The tested EOs at 48 h showed only mild toxicity until 0.1 mg/mL (from 12.5 to 35.0% for the pulegone/isomenthone chemotype). As expected, at the highest concentration tested (1 mg/mL) MPLI and MPFI showed the highest toxicity (95.0%), followed by MPLIII and MPFIII (75.0 and 50.0%, respectively), MPLII and MPFII (~30.0%). This is the first study, which investigates the toxicity of the *M. pulegium* EOs on *Artemia salina* and no data are currently available on the eco-compatibility of these EOs. However, the present results demonstrate once again that there is a direct correlation between pulegone content and toxicity, as already demonstrated in arthropods and mammals [40,41].

In conclusion, it is possible to speculate that the EOs tested are safe at low concentrations (0.0001-0.1 mg/mL) for aquatic organisms and could be used safely in agriculture as eco-sustainable bio-herbicides.

## 3. Materials and Methods

### 3.1. Standards and Reagents

Starch azure, porcine pancreatic amylase, calcium chloride, acetic acid, hydrochloric acid, acetone, dimethyl sulfoxide (DMSO), dichloromethane (CH_2_Cl_2_), and potassium bichromate (K_2_Cr_2_O_7_) were purchased from Sigma Aldrich (Milan, Italy).

### 3.2. Plant Material and Sample Preparation

*M. pulegium* plants were harvested in June-July 2019 in three different areas of Sicily (Italy): Isola Lunga (Marsala, TP), Castellana Sicula (Palermo), and Castronovo di Sicilia (Palermo) (Figure 1). Prof. Francesco Maria Raimondo carried out the taxonomic identification. Details of *M. pulegium* samples are shown in Table 4.

Once in the laboratory, the plants were immediately processed by manually separating flowers (MPF) and leaves (MPL), and 50 g of each were subjected to hydrodistillation by a Clevenger apparatus [42] until no significant increase in the EOs were observed (3 h).

The EOs were dried on Na_2_SO_4_ and stored in sealed vials with nitrogen headspace in the dark until analyses. EOs were diluted in dichloromethane (10% *v*/*v*) for phytochemical analyses. On the contrary, stock solutions in acetone or DMSO were prepared to test the biological activities.

### 3.3. Micromorphological Analyses

Micromorphological features of mature leaves and flowers of *M. pulegium* plants collected in three different areas of Sicily were studied by light (LM) and scanning electron microscopy (SEM).

Epidermal peels and transversal sections of these organs were made using a razor blade and dissecting forceps. Samples were then mounted in distillate water and observed under a Leica D.M. 2000 microscope equipped with a digital camera (DFC 320, Leica Microsystems, Wetzlar, Germany).

For SEM analyses samples of leaves (1.5–2.0 cm^2^) and entire flowers were fixed in 70% ethanol-FineFix working solution (Milestone s.r.l., Bergamo, Italy) for 24 h at 4 °C, and then dehydrated through ethanol series: 80, 90, 95, and 100% [43]. Subsequently, samples were critical point dried in CO_2_ by a critical point dryer (CPD, K850 2M Strumenti s.r.l., Rome, Italy), mounted on aluminum stubs using glued carbon tabs, and sputter-coated with 10 nm gold. Finally, samples were observed with a Vega3 Tescan LMU SEM (Tescan USA Inc., Cranberry Twp, PA, USA) at an accelerating voltage of 20 kV. For the determination of densities of peltate trichomes on both leaf epidermis and calyx, printed micrographs were used, and measures were repeated three times, according to the method reported by Karray-Bouraoui et al. [20]. The diameter of the glandular heads and the number of secretory cells inside the heads were measured for each sample (MP-I, MP-II, and MP-III) in both leaves and flowers, on at least 10 mature peltate trichomes, by computer-assisted image analysis.

### 3.4. Phytochemical Characterization

Phytochemical analyses were carried out by a GC (7890A) equipped with FID and MS detectors (5975C) (Agilent Technologies Santa Clara, CA, USA). Chromatographic separation was carried out using a HP-5MS capillary column (30 mm, 0.25 mm, 0.25 µm) and helium as carrier gas (1 mL/min). EOs injection (1 µl, 10% in CH_2_Cl_2_, *v*/*v*) was done in split mode (50:1). The injector temperature was 250 °C, whereas detector temperatures were 280 °C and 180 °C for FID and MS, respectively. For GC-MS analysis, the ionization voltage, the electron multiplier, and the ion source temperature were set at 70 eV, 900 V, and 230 °C, respectively. The following elution program was used: 60° C for 6 min increased to 270 °C at 3 °C/min, and held at 270 °C for 4 min [44].

Compounds were identified by calculating their Kovats retention index with respect to the reference n-alkanes C7–C40 mix, matching the mass spectral data with NIST 08 MS library [45], by comparing the MS fragmentation patterns with literature data [46], and by co-injection with commercially available standards. Quantification was carried out by extrapolation of the compound’s peak areas from GC-FID profiles.

### 3.5. Phytotoxic Activity

The phytotoxic activity was evaluated on germination and radical elongation of several species: *R. sativus* L., *L. sativa* L., *L. sativum* L., *S. lycopersicum* L., *L. multiflorum* Lam., and *P. oleracea* L. These seeds are often used for their easy and well-known germinability.

*R. sativus*, *L. sativa*, *L. sativum*, and *S. lycopersicum* seeds were purchased from Blumen group SRL (Emilia-Romagna, Italy). *L. multiflorum* seeds were purchased from Fratelli Ingegnoli Spa (Milano, Italy), and *P. oleracea* seeds from W. Legutko SRL (Jutrosin, Poland). The seeds were sterilized in 95% ethanol for 15 s and sown in Petri dishes (Ø = 90 mm), on three layers of Whatman filter paper. They were impregnated with 7 mL of deionized water used as first control to verify the germinability of the seeds, 7 mL of water–acetone mixture (99.5:0.5, *v*/*v*) as second control because EOs were dissolved in this mixture for their lipophilicity, or 7 mL of the tested solution at different doses (100, 10, 1 and 0.1 µg/mL). Controls, carried out with water–acetone mixture alone, showed no appreciable differences in comparison with controls in water alone. The germination conditions were 20 ± 1 °C, with a natural photoperiod. Seed germination was observed in Petri dishes every 24 h. A seed was considered germinated when the protrusion of the root became evident [47]. On the fifth day (after 120 h), the effects on radicle elongation were measured in cm. Each determination was repeated three times, using Petri dishes containing 10 seeds each. Data are expressed as the mean standard deviation for both germination and radicle elongation.

### 3.6. Alpha-Amylase Inhibitory Assay

Alpha-amylase inhibition assay was carried out according to the previous method [48] with slight modification. Briefly, starch azure (10%) and α-amylase (2 U/mL) were suspended by 0.5 M Tris-HCl buffer (pH = 6.9 containing 0.01 M calcium chloride) and boiled for 10 min. Samples were dissolved in 0.1% of DMSO in order to obtain concentrations of 500, 100, 50, 10, and 1 µg/mL. Each sample concentration, 200 µL of starch solution and 100 µL of α-amylase solution were mixed and incubated at 37 °C for 10 min. Then, the reaction was terminated by adding 500 μL of acetic acid solution (50%). The reaction mixture was then centrifuged at 2000 rpm for 5 min. The absorbance of the resulting supernatant was measured at 595 nm using a spectrophotometer (Thermo Scientific Multiskan GO, Monza, Italy). Acarbose was used as a positive control. The experiments were repeated thrice using the same protocol.

Finally, the α-amylase inhibition activity was calculated as the following formula:

(1) $$\alpha -amylase\;inhibition\;activity\;\left(\%\right)=\;\frac{Abs1}{Abs2}\times 100$$

where *Abs1* was the absorbance of sample solution mixed with amylase solution and soluble starch solution, and *Abs2* was the absorbance of the control (sample solution mixed with soluble starch solution).

### 3.7. Brine Shrimp Lethality Assay

In order to investigate the toxicity of the EOs, brine shrimp (*Artemia salina*) bioassay was carried out according to Caputo et al. [49]. Brine shrimp’s eggs were purchased by a local pet shop, placed in a hatcher chamber containing artificial seawater, and incubated for 48 h at RT with continuous aeration and illumination. Stock solutions of *M. pulegium* EOs (0.01–1000 µg/mL) and K_2_Cr_2_O_7_ (50 mg/mL) as a positive control, were prepared in DMSO. After that, 20 µL of each sample/control was seeded in a 24-well plate diluting 1:1000 *v/v* in artificial seawater (0.1% DMSO). Ten nauplii per well were added and incubated for 48 h in the same conditions reported above.

Surviving larvae without abnormal swimming behavior were counted after 24 h and 48 h by a stereomicroscope (SMZ-171 Series, Motic). Two negative controls (10 larvae treated with artificial seawater containing 0.1% DMSO and 10 larvae treated with artificial seawater only) were also evaluated. Three independent experiments (*n* = 10) were carried out for each treatment. Lethality was calculated using the following equation:

(2) $$\%\;Lethality=\;100- [\left(slt\times 100\right)]/slc$$

where *slt* were the survival larvae treated with *M. pulegium* EOs or K_2_Cr_2_O_7_, whereas *slc* were the survival larvae treated with artificial seawater with 0.1% DMSO (negative control).

### 3.8. Statistical Analysis

Results were expressed as mean ± standard deviation (S.D.) of three independent experiments (*n* = 3 for micromorphological, phytochemical, and phytotoxic analyses and *n* = 10 for brine shrimp lethality assay) and analyzed by one-way analysis of variance (ANOVA) by GraphPad Prism 6.0 (Software Inc, San Diego, CA, USA). Dunnett’s multiple comparisons test was used for phytotoxicity and brine shrimp lethality assays, whereas Tukey’s test was used for processing the phytochemical data. *T*-test with Bonferroni’s correction was used for processing the micromorphological data. Results were considered significant for *p* < 0.05.

## 4. Conclusions

This is the first study which investigated the micromorphology, phytochemical profile, phytotoxic activity and eco-compatibility of EOs obtained from *M. pulegium* leaves and flower coming from different areas of Sicily.

Our micromorphological analyses show few significant differences in dimension and abundance of the trichomes of plants from the different Sicilian sites. The higher salinity of the clayey-saline depressions where MP-I grows, in comparison with the soil of MP-II and MP-III, does not significantly affect peltate trichome density and distribution. Moreover, the complex of morphological and phytochemical data indicate that soil salinity influences the composition of the essential oil, rather than its yield.

Indeed, the phytochemical comparison between the EOs coming from the three different Sicilian sites highlighted a very particular chemical composition allowing the identification of two different chemotypes, pulegone/isomenthone for MP-I and piperitone/isomenthone for MP-II and MP-III, demonstrating that the pedo-climatic features strongly affect the expression of the toxic metabolite pulegone.

*M. pulegium* EOs showed selective phytotoxic properties associate often with the inhibition of α-amylase activity, one of the enzymes involved in the seed growth regulation. However, the piperitone/isomenthone chemotype (MP-II and MP-III) showed the best phytotoxic activity both against the germination and the radical elongation of the crop species investigated in the present study.

Finally, the mild and dose-dependent toxicity found on *Artemia salina* corroborates that there is a direct correlation between pulegone expression and ecotoxicity as already demonstrated previously in arthropods and mammals.

These findings open new perspectives for safe uses, in particular of the piperitone/isomenthone chemotype of this plant species, providing a rationale for the possible use of *M. pulegium* EOs as eco-friendly bio-herbicides.

## Figures and Tables

**Table 1:** Peltate trichome density and diameter, and essential oil (EO) yield, of the leaf and flower (calyx) of *Mentha pulegium* (MP), collected at three different locations in Sicily (MP-I, MP-II, and MP-III).

		MP-I	MP-II	MP-III
**Density (n/mm^2^)**	leaf ad. s.	5.0 ± 1.6^#^ (3)	2.6 ± 1.0^#^ (3)	3.6 ± 0.4^#^ (3)
	leaf ab. s.	21.6 ± 1.8^#^ (3)	18.1 ± 3.7^#^ (3)	15.9 ± 4.3^#^ (3)
	fl. calyx	13.7 ± 2.8* (3)	22.1 ± 1.6* (3)	19.0 ± 1.6 (3)
**Diameter (μÌm)**	leaf	87.2 ± 3.4*^#^ (16)	86.4 ± 4.5^#^ (15)	83.0 ± 3.2^*^^#^ (15)
	fl. calyx	100.1 ± 3.7^#^ (10)	101.7 ± 4.0^#^ (10)	104.5 ± 10.2^#^ (10)
**EO yield (*w*/*w* %)**	leaf	3.2	2.7	2.9
	flower	2.8	2.7	2.6

Data of peltate trichome density and diameter are expressed as mean ± S.D. (*n* = 3). Asterisks (*) indicate groups significantly different from each other in horizontal comparisons (*p* < 0.05), and hashes (#) in vertical comparisons (*p* < 0.01). ad. s. = adaxial surface; ab. S = abaxial surface; fl. = flower.

**Table 2:** Chemical composition of *Mentha pulegium* EOs collected in three different Sicilian locations (I, II, and III; see Section 3.2 for more details; L= leaf; F=flower). Bold values refer to the most abundant compounds identified. Results are expressed as mean area percentage (%) ± standard deviation (S.D.) of three independent determinations in triplicate (*n* = 3).

	KI^a^	Identification^b^	MPLI	MPFI	MPLII	MPFII	MPLIII	MPFIII
2.5-diethyl tetrahydrofuran	884	1,2	-	t	-	-	t	-
Santolina triene	908	1,2	-	-	-	-	t	-
α-Thujene	924	1,2,3	-	t	-	t	t	-
α-Pinene	939	1,2,3	0.1 ± 0.0*	0.3 ± 0.0*	0.2 ± 0.0	0.2 ± 0.0	0.2 ± 0.0	t
Camphene	954	1,2,3	-	t	-	t	t	-
Thuja-2,4(10)-diene	960	1,2	-	-	-	-	t	
β-Thujene	968	1,2,3	t	-	0.3 ± 0.0*	0.1 ± 0.0*	t	-
Sabinene	975	1,2,3	-	t	0.2 ± 0.0^§^	0.2 ± 0.0^§^	0.2 ± 0.0^§^	t
β-Pinene	979	1,2,3	0.1 ± 0.0*	0.3 ± 0.0*	-	-	-	-
3-Octanone	983	1,2	0.1 ± 0.0*	t	-	t	-	-
Myrcene	990	1,2,3	0.1 ± 0.0	0.3 ± 0.0*	0.1 ± 0.0	0.2 ± 0.0*	0.1 ± 0.0	-
3-Octanol	995	1,2	1.1 ± 0.1*	0.9 ± 0.0*	0.8 ± 0.0	0.8 ± 0.1	0.2 ± 0.0*	-
α-Phellandrene	1002	1,2	-	-	-	t	t	-
p-Mentha-1(7),8-diene	1004	1,2	t	-	-	-	-	-
t-Butyl Benzene	1008	1,2	-	t	-	-	-	-
δ-Carene	1011	1,2,3	-	t	-	-	-	-
α-Terpinene	1017	1,2,3	-	-	-	t	-	-
1,2,3,4-Tetramethylbenzene	1027	1,2	t	-	-	-	-	
Limonene	1029	1,2,3	1.7 ± 0.1	2.3 ± 0.1*	1.8 ± 0.1	3.6 ± 0.2*	0.3 ± 0.0*	t
1.8-Cineole	1031	1,2,3	0.1 ± 0.0*	t	0.1 ± 0.0	0.1 ± 0.0	t	-
(Z)-β-Ocimene	1037	1,2	t	t	-	t	-	-
γ-Terpinene	1059	1,2,3	t		-	-	t	-
Terpinolene	1088	1,2,3	t	t	-	t	t	-
trans-Sabinene hydrate	1098	1,2	t	t	-	t	t	-
*cis*-Thujone	1102	1,2					t	
3-Octanol acetate	1123	1,2	0.2 ± 0.0*	-	-	-	-	-
Menthone	1152	1,2,3	0.8 ± 0.0°	0.8 ± 0.0°	0.2 ± 0.0	0.2 ± 0.0	0.8 ± 0.0°	0.3 ± 0.0*
**Isomenthone**	1162	1,2,3	34.0 ± 1.1*	30.9 ± 1.4*	**7.2 ± 0.3**	5.3 ± 0.3*	13.0 ± 0.9*	**8.0 ± 0.3**
Menthol	1171	1,2,3	1.0 ± 0.1*	1.2 ± 0.0*	-	t	0.5 ± 0.0*	-
*iso*-Menthol	1182	1,2	1.3 ± 0.1*	0.4 ± 0.0*	-	t	t	-
α-Terpineol	1188	1,2,3	-	-	-	t	0.2 ± 0.0	-
Isopulegone	1229	1,2	0.1 ± 0.0^¥^	t	-	-	0.1 ± 0.0^¥^	-
**Pulegone**	1237	1,2,3	**56.8 ± 2.2** ^Φ^	**60.2 ± 1.9** ^Φ^	-	-	0.2 ± 0.0*	-
(2*Z*)-hexenyl isovalerate	1244	1,2	-	-	-	t	-	-
(2*E*)-hexenyl valerate	1247	1,2	-	-	t	t	-	-
**Piperitone**	1252	1,2,3	0.5 ± 0.0*	0.7 ± 0.0*	**88.5 ± 2.3**	**88.0 ± 2.9**	80.3 ± 1.7*	**89.4 ± 1.6**
*cis*-Carvone oxide	1263	1,2	t	-	-	-	-	-
*neo*-Menthyl acetate	1272	1,2	0.1 ± 0.0*	-	-	-	-	-
*trans*-Carvone oxide	1276	1,2	t	-	-	-	0.2 ± 0.0	-
*neo*-Menthyl acetate	1278	1,2	0.4 ± 0.0*	t	-	-	0.1 ± 0.0*	-
*iso*-Menthyl acetate	1305	1,2	0.6 ± 0.0*	0.8 ± 0.0*	-	0.3 ± 0.0*	2.9 ± 0.1*	1.9 ± 0.1*
α-Copaene	1376	1,2,3	t	t	-	t	t	-
β-bourbonene	1385	1,2	t	-	t	t	t	-
β-Cubebene	1388	1,2	t	t	-	-	-	-
(*E*)-Caryophyllene	1419	1,2,3	0.1 ± 0.0*	t	0.2 ± 0.0	0.2 ± 0.0	0.2 ± 0.0	0.1 ± 0.0*
β-Copaene	1432	1,2,3	t	t	-		-	-
α-Humulene	1454	1,2,3	0.1 ± 0.0*	t	0.2 ± 0.0	0.1 ± 0.0	0.1 ± 0.0	t
Germacrene D	1481	1,2	0.4 ± 0.0*	0.3 ± 0.0*	t	t	t	-
α-selinene	1498	1,2	-	t	-	-	-	-
δ-Cadinene	1523	1,2,3	t	t	-	-	-	-
α-Cadinol	1654	1,2,3	t	-	-	-	-	-
(*Z*.*Z*)-Farnesyl acetone	1860	1,2	-	t	-	-	-	-
Monoterpene hydrocarbons Monoterpenes oxigenated Sesquiterpene hydrocarbons Others			2.2	3.4	2.6	4.4	0.9	0.2
			92.2	92.8	96.0	93.7	94.5	97.7
			0.8	0.4	0.5	0.4	0.4	0.2
			4.8	3.5	0.9	1.5	4.2	1.9

^a^ Linear retention index on a HP-5MS column; ^b^ Identification method: 1 = linear retention index; 2 = identification based on the comparison of mass spectra; 3 = Co-injection with standard compounds; t = traces, less than 0.01%; -, not found; MPL, M. pulegium leaves; MPF, M. pulegium flowers. * *p* < 0.001 vs. all other EOs; ^§^ *p* < 0.001 vs. MPLI, MPFI and MPFIII; ° *p* < 0.001 vs. MPLII and MPFII; ^¥^ *p* < 0.001 vs. MPFI, MPLII, MPFII, MPFIII; ^Φ^ *p* < 0.001 vs. MPLII, MPFII, MPFIII.

**Table 3:** α-Amylase inhibitory activity of EOs from the three sites (MP-I, MP-II, MP-II) (L = leaf; F = flower)

Essential Oils	IC_50_ Values (µg/mL)
MPLI	662.8 ± 5.8
MPFI	785.3 ± 7.1
MPLII	N.A.
MPFII	1076.7 ± 8.9
MPLIII	830.9 ± 6.3
MPFIII	638.9 ± 9.1
Acarbose (positive control)	109.3 ± 5.6

N.A. = not active.

**Table 4:** Details of *M. pulegium* leaf (MPL) and flower (MPF) samples.

Collection Site	GPS Coordinates	Site Features	Voucher Specimens
**I**: Isola Lunga (TP)	37°54′06″ N, 12°27′07″ E	Clayey-saline depressions	R&Sp * 05/19
**II**: Castronovo di Sicilia (PA)	37°40′09″ N, 13°38′54″ E	Clayey-moist soil	R&Sc ^§^ 06/19
**III**: Castellana sicula (PA)	37°45′35″ N, 13°59′35″ E	Clayey-moist soil	R&Sc ^§^ 07/19

* R&Sp, Raimondo & Spadaro; ^§^ R&Sc, Raimondo & Schimmenti.

## Data Availability

The data presented in this study are available on request from the corresponding author.

## Supplementary Materials

The following are available online, Figure S1: Phytotoxic activity of *M. pulegium* leaves (II) EO against radical elongation of *S. lycopersicum* (A), *L. sativa* (B), *L. sativum* (C), *R. sativus* (D), *L. multiflorum* (E) and *P. oleracea* (F) 120 h after sowing. Results are the mean of three experiments ± standard deviation; Figure S2: Phytotoxic activity of *M. pulegium* leaves (II) EO against germination of *S. lycopersicum* (A), *L. sativa* (B), *L. sativum* (C), *R. sativus* (D), *L. multiflorum* (E) and *P. oleracea* (F) 120 h after sowing. Results are the mean of three experiments ± standard deviation.

## Sample Availability

Samples of the essential oils are available from the authors.
