# Supplementary material for: Mentha pulegium L.: A Plant Underestimated for Its Toxicity to Be Recovered from the Perspective of the Circular Economy
Source: Molecules. 2021 Apr 8;26(8):2154. doi: 10.3390/molecules26082154 (PMC8069592; doi:10.3390/molecules26082154)
Supplement: Supplementary file 1 [file molecules-26-02154-s001.pdf]

Supplementary material

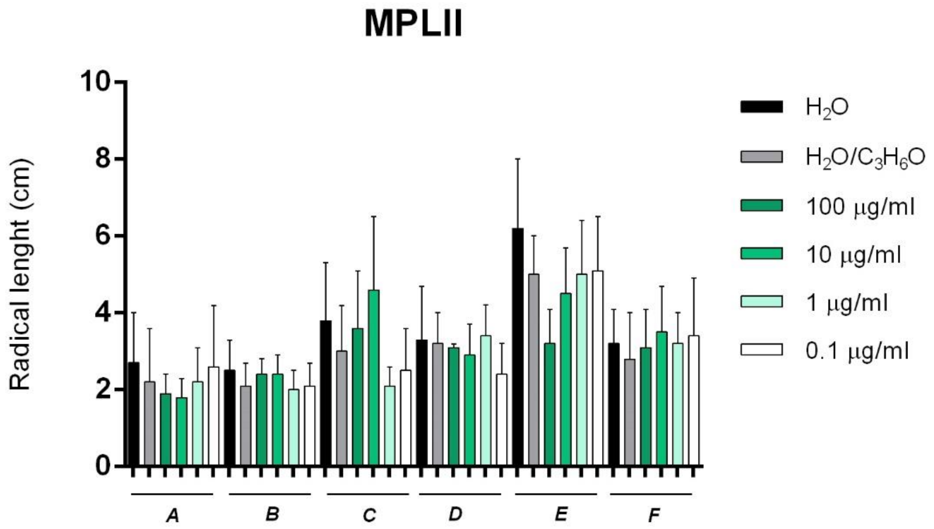

Figure S1. Phytotoxic activity of *M. pulegium* leaves (II) EO against radical elongation of *S. lycopersicum* (A), *L. sativa* (B), *L. sativum* (C), *R. sativus* (D), *L. multiflorum* (E) and *P. oleracea* (F) 120 h after sowing. Results are the mean of three experiments ± standard deviation.

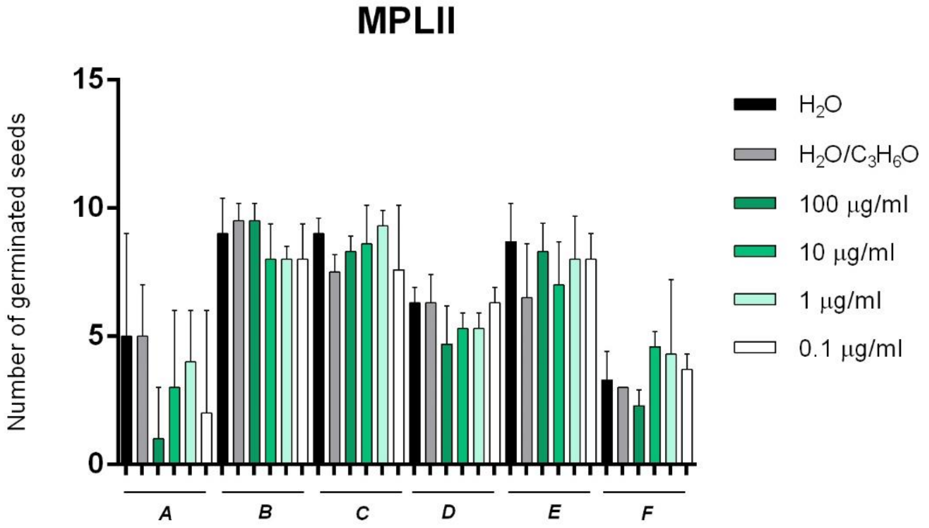

Figure S2. Phytotoxic activity of *M. pulegium* leaves (II) EO against germination of *S. lycopersicum* (A), *L. sativa* (B), *L. sativum* (C), *R. sativus* (D), *L. multiflorum* (E) and *P. oleracea* (F) 120 h after sowing. Results are the mean of three experiments ± standard deviation.
